# Evolution through cold and deep waters: the molecular phylogeny of the Lithodidae (Crustacea: Decapoda)

**DOI:** 10.1007/s00114-018-1544-2

**Published:** 2018-02-27

**Authors:** Sally Hall, Sven Thatje

**Affiliations:** Ocean and Earth Science, National Oceanography Centre, University of Southampton, European Way, Southampton, SO14 3ZH UK

**Keywords:** King crab, Radiation, Phylogeny, Mutation rate, Speciation

## Abstract

The objectives of this work are to use gene sequence data to assess the hypothesis that the Lithodinae arose from ancestors with uncalcified abdomens in shallow waters of the North-East Pacific, investigate the monophyly and interrelationships of genera within the Lithodinae and to estimate the scale and minimum number of biogeographic transitions from the shallow environment to the deep sea and vice versa. To do this, phylogenetic analysis from three mitochondrial and three nuclear markers was conducted using minimum evolution, maximum likelihood and Bayesian methods. The Lithodinae as defined to include North Pacific genus *Cryptolithodes* may be paraphyletic, with the Hapalogastrinae and *Cryptolithodes* as sister taxa. This implies that the soft-bodied abdomen of the Hapalogastrinae might not be plesiomorphic for the Lithodidae. *Paralomis*, *Lopholithodes*, *Phyllolithodes*, *Lithodes* and *Neolithodes* share a common ancestor, from which the North Pacific Hapalogastrinae did not descend. Lithodid ancestors are likely to have had a north Pacific, shallow water distribution and to have had planktotrophic larvae. North Pacific genus *Paralithodes* is paraphyletic; *P. brevipes* is the most basal member of the genus (as sampled) while *P. camtschaticus* and *P. platypus* are more closely related to the genera *Lithodes* and *Neolithodes*. Genera *Lithodes*, *Neolithodes* and *Paralomis* (as sampled) are monophyletic if *Glyptolithode*s is included within *Paralomis*. *Lopholithodes* is closely related to, but not included within, the *Paralomis* genus. *Paralomis* is divided into at least two major lineages: one containing South Atlantic, West African, and Indian Ocean species, and the other containing Pacific and South American species. Several species of *Paralomis* do not resolve consistently with any other groups sampled, implying a complex and possibly rapid global evolution early in the history of the genus. Relationships within the *Lithodes* genus vary between analytical methods, suggesting that conclusions may not be stable. Consistently, however, Indian Ocean and Pacific forms—*L. murrayi*, *L. longispina* and *L. nintokuae* form a group separated from Atlantic species such as *L. santolla*, *L. confundens*, *L. maja* and *L. ferox*.

**Electronic supplementary material:**

The online version of this article (10.1007/s00114-018-1544-2) contains supplementary material, which is available to authorized users.

## Introduction

### The anomuran Lithodidae

The anomuran crab family Lithodidae Samouelle 1819 comprises a great diversity of morphological and ecological forms: from abyssal crabs with walking legs longer than a metre to intertidal forms such as the genus *Cryptolithodes* Brandt 1848, which has tiny legs covered by a laterally expanded carapace (Bowman 1972; see also Hall and Thatje 2010). In the deep sea, the large subfamily Lithodinae includes species occupying hydrothermal vent environments (de Saint Laurent and Macpherson 1997), as well as species amongst the few known ‘reptant’ decapods from the Southern Ocean (Thatje and Arntz 2004; Aronson et al. 2015). Study of the origins of the Lithodidae has increased in recent decades (Zaklan 2002; Hall and Thatje 2009; Bracken-Grissom et al. 2013), with particular interest being placed on the putative relationship between ‘primitive’ (morphologically less derived) lithodids and hermit crabs of the family Paguridae (Bracken-Grissom et al. 2013; Noever and Glenner 2017). The Lithodidae have an enigmatic evolutionary history. Molecular evidence indicates a strong and recent (13–25 Ma BP) relationship between fully carcinised lithodids and shell-dwelling members of the genus *Pagurus* (Cunningham et al. 1992). From this, it has been hypothesised that the uncalcified abdomen uniting the subfamily Hapalogastrinae reflects the retention of a primitive condition, and that primitive groups from the shallow north Pacific were the seeding populations for the global deep-water expansion of the Lithodinae. This hypothesis remains a source of controversy (McLaughlin et al. 2007).

Here, we investigate the process of global radiation in groups of deep-sea Lithodinae (Bouvier 1895; Makarov 1938): a topic that has not specifically been addressed since the advent of cladistic systematics (Hennig 1966). This will be done by (i) presenting a molecular phylogeny to investigate the origins of the deep-sea Lithodinae from within the Lithodidae, (ii) elucidating phylogenetic relationships within deep-sea lithodine genera *Paralomis* and *Lithodes* as case studies of deep-sea radiations and (iii) by comparing potential geographical and physiological boundaries with the present distribution of the deep-sea Lithodinae and their shallow-water relatives in South America.

## Materials and methods

### Sample origin

Tissue samples were obtained from several sources in order to get a wide coverage and high number of replicates of lithodid species. Egg and dactylar tissue of the walking leg from preserved museum specimens were obtained with permission from Natural History Museum, London; Senckenberg Museum, Frankfurt; Musée National d’Histoire Naturelle, Paris; Institut de Ciencies del Mar, Barcelona; US National Museum of Natural History, Smithsonian Institute, Washington; collections in Chile and Miami; CADIC, Ushuaia, Argentina and the ‘Discovery Collection’, National Oceanography Centre, Southampton. Tissue from fresh specimens was obtained from Spanish cruises around Mauritania (2008), French fishing vessels near Kerguelen (2008), ROV Isis from on board RRS James Clark Ross (JCR167), fisheries and dives around the Falklands and South Georgia and Norwegian commercial fishing vessels.

### Species sampling

To elucidate worldwide relationships between species of Lithodinae, the sampling aim was to obtain molecular sequences from a wide range of lithodid species (Online Supplementary Table 1). Although obtaining specimens was not a limitation, the difficulty of obtaining non-fragmented DNA from preserved deep-sea samples led to reduced (and slightly unpredictable) success. Approximately 30% of all DNA samples were extracted from ethanol-preserved or frozen tissue and 70% from formalin-preserved specimens. Including sequences obtained from the NCBI GenBank nucleotide database (GenBank 2017), 16/61 *Paralomis*, 9/21 *Lithodes* and 3/10 *Neolithodes* species were used in this study, covering approximately one quarter of the species known to exist worldwide. In addition, sequences were obtained from NCBI GenBank for some genes of *Cryptolithodes* (two species), *Hapalogaster* (two species), *Oedignathus*, *Lopholithodes* (two species), *Glyptolithodes*, *Phyllolithodes*, *Paralithodes* (three species) and non-lithodid genera *Aegla*, *Pagurus*, *Emerita*, and *Lomis*.

### Gene targets

Appropriate genetic targets for family level phylogenetic analysis are those that mutate quickly enough for differences to be observed between taxa, but not so quickly that they diverge substantially within an interbreeding population. Targeting a gene where there is no detailed prior knowledge of the organism’s genome can be done using universal primers that anneal with highly conserved regions of functional copies of the gene (Palumbi and Metz 1991; Folmer et al. 1994). Two sections of the nuclear small subunit ribosomal gene (18S), and three sections of nuclear large subunit rDNA (28S) were examined because of a high rate of sequence divergence observed in the expansion segments of related organisms (Nelles et al. 1984; Crease and Taylor 1998; Held 2000). The ‘internally transcribed spacer 1’ (ITS1) is a region of non-translated DNA which is transcribed along with functional rRNA genes in eukaryotes. Its principally nonstructural role means that it is largely free from conservative selection and can be particularly useful for phylogenetic analysis of closely related groups, particularly decapods (Chu et al. 2001; Armbruster and Korte 2006).

### 28S amplicons

Several sets of sequences were available for parts of the anomuran 28S gene on the NCBI GenBank nucleotide database. The longest stretches of lithodid DNA, covering 2478, 2473 and 2474 bp, respectively, are from *Lithodes santolla* (GenBank AY596100.1), *Paralithodes camtschatica* (GenBank AB193824.1) and *Paralithodes platypus* (GenBank AB193821.1). Lithodid genes from two additional genera (*Neolithodes brodiei* ×2, *Paralomis formosa*, *Paralomis elongata*) were sequenced in order to supplement an alignment of *L. santolla* and *Paralithodes* for a preliminary analysis of sequence divergence. The lithodid 28S gene was amplified in three sections in order to probe for levels of inter-specific variation at different locations on the gene. Based on the results of this preliminary analysis, the Lsp28SBF and 28SBR primers were used to amplify a partial 28S sequence from a wider lithodid taxon set.

### 18S amplicons

The 1830 bp of the *L. santolla* 18S gene (GenBank AF439385.1) and 14 other anomuran sequences of similar length were obtained from the NCBI GenBank database. Comparison with the 18S secondary structure of *Drosophila melanogaster* in Hancock et al. (1988) shows that this fragment contains the V2 and V4 expansion segments of the molecule. Primers 18A1 and 1800R amplify a theoretical 1800 bp of the gene. V4for and V4rev target 246 bp of in-group DNA, to ensure that a double-stranded section (where forward and reverse sequences overlap) of this variable region is amplified in all samples. To investigate the suitability of the 18S gene for phylogenetic studies in Lithodidae, the genes from four species (*N. brodiei* ×2, *P. formosa*, *P. elongata*) were sequenced for comparison with *L. santolla*.

### Tissue sampling protocols

Wherever fresh tissue was obtained for this study, the samples were either frozen whole, or sampled in semi-sterile conditions into cold (0 °C) 70% ethanol as soon as possible after death. Frozen tissue, after sampling, was stored in ethanol at 4 °C, since repeated freezing and thawing of tissue is detrimental to the structure of DNA (Shikama 1965). Approximately 1.5 mm^3^ muscular tissue from one of the elements of the walking legs was sampled through a small incision in the arthrodial membrane. On some occasions, it was only possible to obtain material from gill tissue or from eggs. The success of amplification from embryonic tissue was lower than for muscular tissue and especially so in formalin-preserved samples. Depending on the method of DNA extraction, the tissue was placed directly into the critical-point drying protocol, or into a Qiagen buffer with proteinase K, which digests the tissue and prevents any autolytic processes.

### Methods for well-preserved samples

Tissue was placed in 20 μl proteinase K, buffered with 180 μl ATL and incubated at 55 °C for 1–8 h according to the instructions of the Qiagen DnEasy Blood and Tissue kit protocol. Selective filtration of the resulting DNA solution was done using Qiagen centrifuge columns, and DNA is eluted after two wash steps into AE buffer for storage or PCR. All sample extractions were repeated once.

### Methods for sub-optimally preserved samples

Samples taken from wet-preserved museum specimens, including those for which the Qiagen columnar filtration system isolated DNA, underwent additional extraction using a method based on critical-point drying (Palero et al. 2010). In order to reduce costs and provide a similar effect to that proposed in (Fang et al. 2002), this study used tetramethylsilane (TMS) as a strong dehydrating agent, which maintains tissue structure (Ubero-Pascal et al. 2005; Palero et al. 2010). Muscle from the dactylus, was extracted into 50–100 μl TMS solution and incubated for 1 h with gentle agitation so that the tissue absorbed the dehydrating agent. The cap of the tube was then opened within a sterile laminar flow cabinet to let the TMS evaporate. Dehydrated tissue was transferred to a 1.5-ml Eppendorf tube with 200 μl of 10% Chelex solution in pH 8.0 TE buffer and 20 μl of proteinase K solution (20 mg/ml). This was incubated for 2–3 h at 55 °C in a thermomixer and then centrifuged for 1 min at 10,000 rpm to separate the Chelex from the supernatant. Cells were heat-shocked at 95 °C for 15 min; 100 μl of supernatant, containing extracted DNA and minimal Chelex, was transferred into a fresh tube; 1–2 μl of this supernatant was used for each 25 μl PCR reaction.

### PCR and cleaning

PCR reactions were conducted using GE Healthcare illustra PuReTaq ready-to-go PCR beads: these contain 2.5 units of PuReTaq DNA polymerase, 10 mM Tris-HCl, (pH 9.0 at room temperature), 50 mM KCl, 1.5 mM MgCl2, 200 μM of each dNTP. Beads are temperature-stable until hydration to a total reaction volume of 25 μl, at which time they were stabilised at 4 °C in an ice bucket. Rehydration mixture consisted of nuclease-free water, 10 pmol each of forward and reverse primer and approximately 50 ng of DNA template in solution (AE or TE buffer). PCR reactions were performed using an Eppendorf Master Cycler with temperature profile as according to the guidelines provided for the PuReTaq polymerase. The results were visualised by staining DNA in a (0.1%) ethidium bromide bath followed by destaining in a distilled water bath. PCR products were separated from the residual primers, polymerase and salts using the Qiagen QIAquick PCR-purification kit, using the manufacturer’s protocol. Dideoxy-chain termination sequencing (Sanger et al. 1977) was performed remotely, either by Eurofins, Germany or Macrogen Inc., Korea.

### Phylogenetic analyses

Total evidence (TE_B_) dataset were created suing gene fragments 16S, COI, 28SB and ITS1; alignments contained 50 unique sequences of length (l) 2110 bp (ambiguous alignment I out-groups or missing data [N] were distinguished from insertion mutations [−]).

In <PAUP*4.0b10> (Swofford 2000), a partition homogeneity test, command name <<HOMPART>> examined whether ME trees generated for the single-gene (partition) datasets [16S, COI, ITS, 28S] varied significantly over 1000 replicates from those produced when the data are combined as TE_B_. This tested the hypothesis that a natural partition of TE_B_ (by genes) was significantly different from a random partition of the dataset.

For each gene and for the combined dataset (TE_B_), the model of evolution most likely to account for the observed sequence diversion was assessed using <Modeltest 3.7> (hLRT).

The phylogenetic signal from each gene was examined by creating ME and ML trees in <Mega3.1> and <PHYLIP*DNAml*>, respectively. For TE_B_, ML, ME and BAY, trees were produced and discussed.

For discussion, a schematic of relationships between there genera was taken from TE_B_ trees to form a single tree, TE_C_. In TE_C_, species with monophyletic genera were condensed to a single taxon label and polyphyletic or paraphyletic genera were indicated by multiple taxon labels. Less frequent alternative topologies were indicated by dotted lines on the same tree.

## Results

### Extraction and amplification

PCR products of expected lengths were produced for approximately 80% of freshly sampled (non-museum) specimens and around 5% of museum specimens preserved using an unknown medium or method. Of the 30 formalin-fixed lithodid tissue samples from which DNA was extracted using critical-point drying (Palero et al. 2010), 18 failed to produce amplicons, 5 produced PCR products but were not successfully sequenced and 7 samples (23%) produced fully sequenced PCR products (GenBank accession numbers: EU493266-EU493270, EU493272-EU493275 and EU493277-EU493278). The results obtained from a database search on GenBank using Megablast (BLASTN v2.2.18) showed that the sequences from formalin-fixed specimens were homologous to the available lithodid sequences. Base frequency homogeneity tests showed no significant variation in base frequencies between taxa for 16S (*p* = 1.000), ITS (*p* = 1.000), 28S (*p* = 0.9988) and COI (*p* = 0.991) datasets.

### COI

**Fig. 1:**
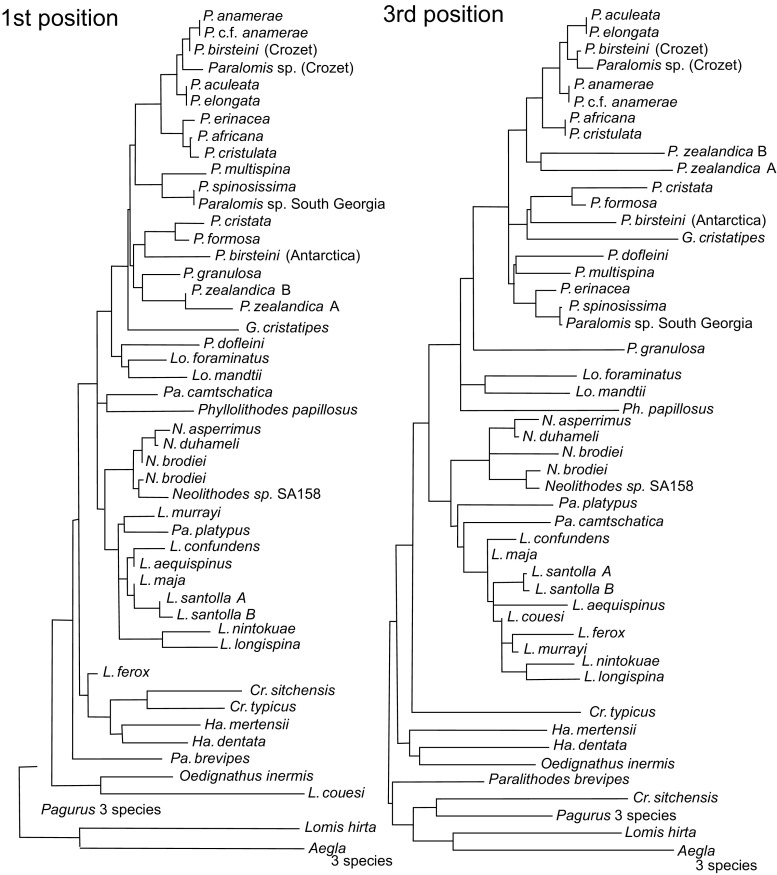
Minimum evolution trees produced in MEGA for COI codon positions 1 and 3. Trees were rooted at the mid-point of the maximum sequence divergence. Abbreviations: N = *Neolithodes*, L = *Lithodes*, P = *Paralomis*, Pa = *Paralithodes*, Lo = *Lopholithodes*, Cr = *Cryptolithodes*, Ha = *Hapalogaster*

Six hundred twenty-one sites were included in the final alignment of the COI gene. The reading frame began on the third position of the alignment and did not include any termination codons. Including changes between the in-group and out-group, 230/621 sites were variable. As would be expected in a protein-coding gene, mutations are heavily biased onto the third position of the codon: there are 39 first position changes, 3 second position changes, and 188 third position changes (Fig. 1).

### 16S

**Fig. 2:**
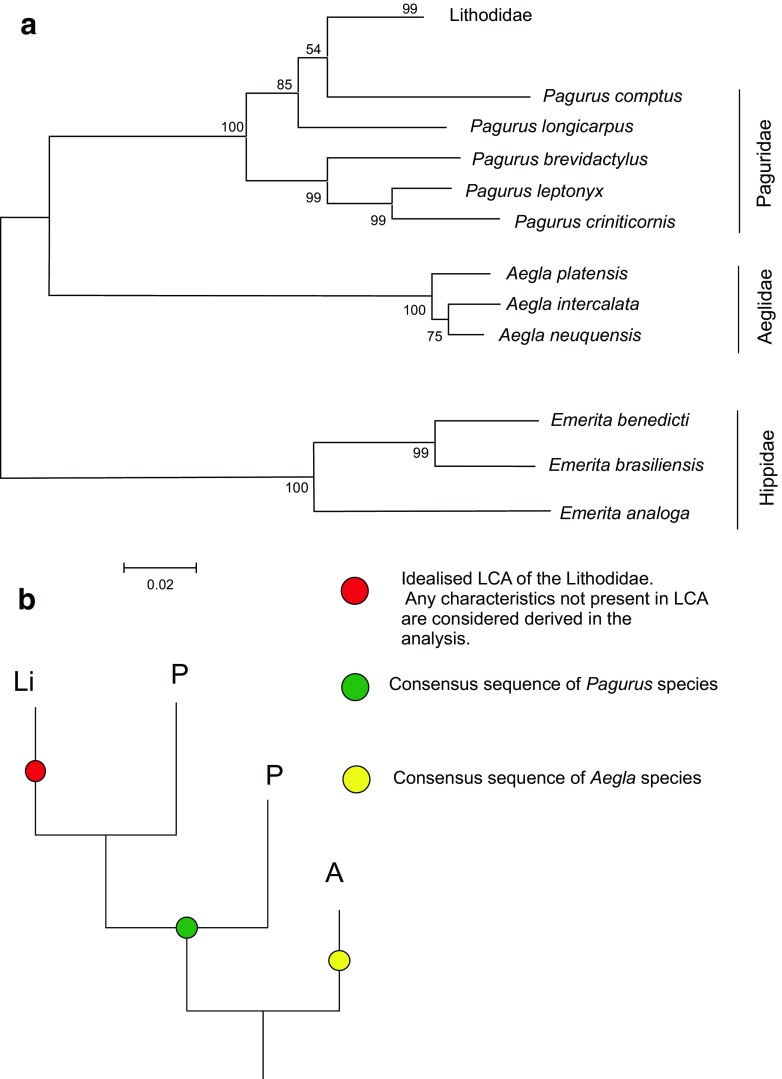
Preliminary investigation of out-groups using the 16S rDNA gene. a AT_A_ tree. A Produced using a close-neighbour interchange algorithm from an initial neighbour joining tree, using the minimum evolution (ME) optimality criterion. Analysis includes all taxa obtained for the 16S rDNA gene, but the lithodid lineage is condensed to a single taxon label. b Schematic of a relationship between Lithodidae (Li), *Pagurus* (P) and *Aegla* (A), and the principle of producing an out-group consensus sequence

The 16s amplicons were sequenced and aligned for 113 specimens. These were used directly in tree ATA (Fig. 2a) and then condensed into 48 consensus sequences, including those for out-group genera *Pagurus* and *Aegla*. Even though rDNA does not have the same reading frame constraints as a protein-coding gene, insertion mutations were absent from the in-group 16S sequences (except two single-base insertions in *Cryptolithodes sitchensis*). At least seven separate regions of nucleotide insertion are present in out-groups, which added some ambiguity to the alignment process in some variable regions.

### COII

Five out of 44 unique lithodid COII sequences had single frame shift mutations and a number of stop codons were produced when translating the alignment. These could have either been introduced through an error in sequencing (the polymerase adding bases stochastically), or could indicate that this is not a functional copy of the gene. No frame shift mutations were seen in the amplified copy of the COI gene: it is possible that these COII primers target analogous versions of the gene and potentially misleading phylogenetic signals are expected. Fifty first position changes, 15 (8 in in-group) second position, and 148 third position changes were seen in the alignment.

### ITS1

Four hundred ninety base pairs of ITS1 were amplified, and there was no ambiguity in the alignment process. Using <Modeltest 3.7>, the model most likely to reflect the molecular evolution of the lithodid ITS1 region was the single substitution rate model (Jukes-Cantor), which specifies equal base frequencies at equilibrium, equal probabilities of transition and transversion mutations and homogenous mutation rates across all sites in the amplified region.

### 18S

In total, three point mutations were observed in lithodids over 1836 bp of 18S rDNA.

The V4 expansion segment was amplified in species from three lithodid genera (*L. santolla*, *P. formosa*, *P. elongata* and *N. brodiei*), but only a single point-mutation was observed. The low level of variation meant that the 18S rDNA gene was not targeted further for analysis.

### 28S

An alignment of 28S gene fragments from 22 anomuran taxa, including 14 species of Lithodidae, indicated low levels of in-group variability in the 5′ region (3/301 bp polymorphic: GenBank AF425344-59, Zaklan 2002a). Gene fragment 28SA includes the D1 expansion segment (Hancock et al. 1988), and polymorphies occurred at 8/739 sites within four lithodine genera (GenBank AY596100.1, AB193824.1, AB193821.1, HM020882-5). Fragment 28SB was variable at 25/683 positions, including base insertions in the sequence of *L. santolla*. Gene fragment NURI had three polymorphic positions in 415 bp of the lithodine genome (GenBank HM020886-9). Sixty specimens produced 30 unique sequences for the 28SB gene fragment, including two out-group consensus sequences (*Pagurus* and *Aegla*). No additional GenBank sequences for in-groups were available in this region, as this appears to be a novel use of these primers within the Lithodidae. The high degree of similarity between in-group sequences meant that the secondary structure of transcribed rRNA could be compared to the conserved arthropod structure. At least six regions of base insertion (or deletion) occur between the out-group and the in-group sequences, indicating a high level of divergence between these sequences in the Anomura.

### Out-groups

(Bootstrap values of tree AT_A_ [Fig. 2a] indicated by *). From the results of an ME analysis of all taxa for the 16S rDNA gene, a consensus of *Aegla* species *A. platensis*, *A. intercalata* and *A. neuquensis* was chosen to root subsequent ML and Bayesian analyses. Tree ATA (Fig. 2a) shows that *Pagurus* species, whilst being close to the monophyletic Lithodidae (*99), were themselves paraphyletic based on the 16S sequence. Results match with a hypothesised scenario (Fig. 2b) in which a consensus sequence of *Pagurus* species should provide a good out-group for this study. Nevertheless, a compromise was made in rooting the trees with *Aegla* species because of the less complicated relationship between them and the Lithodidae. The *Pagurus* consensus sequence was retained in all taxonomic sets and was used as a substitute to root analyses if no corresponding *Aegla* sequence was available. The *Emerita* sequences were particularly different from those of the Lithodidae, implying a distant relationship. *Emerita* was excluded from all other phylogenetic analyses to maximise graphical resolution for taxa within the Lithodidae.

### Genetic variability within the Lithodidae

(Bootstrap values of tree ATA (Fig. 2a) indicated by *). The monophyly of clades within genera *Pagurus* (South American species *P. brevidactylus*, *P. criniticornis*, *P. leptonyx* *99), *Aegla* (*100) and *Emerita* (*100) was confirmed by the production of ME tree ATA for the 16S gene (Fig. 2a). Results for enumeration of polymorphic loci are summarised in Table 1.

**Table 1:** Number of single base polymorphisms between trimmed homologous alignments of parts of five genes (number of species in brackets; N/A = not applicable; Par = *Paralithodes*, Lith = *Lithodes*, Neo = *Neolithodes*, E = *Emerita*)

Clade	COI 631 bp	16S 402 bp	28SB 582 bp	ITS1 490 bp	COII 570 bp	18S
*Pagurus* (3)	N/A	53 (3)	N/A	N/A	N/A	N/A
Lithodidae (40) (10 genera)	219 (40)	68 (45)	N/A	57 (25)	181 (18)	N/A
Lithodinae (35) (7 genera excluding *Cryptolithodes*)	198 (35)	53 (33)	19 (22)	46 (20)	158	3/1960 (3 genera: Par, Lith, Neo)
*Neolithodes* (3)	48 (3)	4 (3)	2 (3)	1 (3)	14 (2)	N/A
*Lithodes* (9)	48 (9)	17 (6)	0 (4)	17 (4)	68 (4)	N/A
*Paralomis* (16)	156 (16)	27 (15)	9 (13)	15 (8)	92 (9)	0/1960 (2)
*Aegla* (3–8)	74 (3)	25 (3)	17 (3)	N/A	N/A	6/1960 (8)
*Emerita* (3)	135 (3)	61 (3)	N/A	N/A	N/A	11/1980 (*E. analoga*, *E. brasiliensis*

### Total evidence trees [TE_B_] = (16S + COI + ITS + 28S)

Over 1000 replicates, ME trees generated for each gene independently did not yield a significantly different phylogenetic signal to that of the combined dataset (*p* = 0.63). This indicates that the data can be combined into a single alignment (TE_B_) without introducing conflicting results. When molecular evidence from fragments of COI, 16S, ITS1 and 28SB were combined, <jModeltest 3.7> predicts the following model of molecular evolution (using hLRT): (HKY + *I* + *G*) is a 2-substitution rate model (Ti/Tv ratio = 3.7305) with unequal base frequencies (*A* = 0.2862, *C* = 0.1995, *G* = 0.1981, *T* = 0.3162), and between-site rate heterogeneity modelled by the Gamma function (shape = 0.3470) and a proportion (0.5318) of invariant sites. For the ME analysis in <MEGA3.1>, the Kimura 2-parameter model of evolution was assumed. For maximum likelihood analysis in <PHYLIPDNAml>, all parameters were taken from the above estimations. For Bayesian analysis, the number of substitution types, (COI = 6, 16S = 2, ITS = 1, 28S = 2) were indicated for each of four gene partitions.

### Genera

**Fig. 3:**
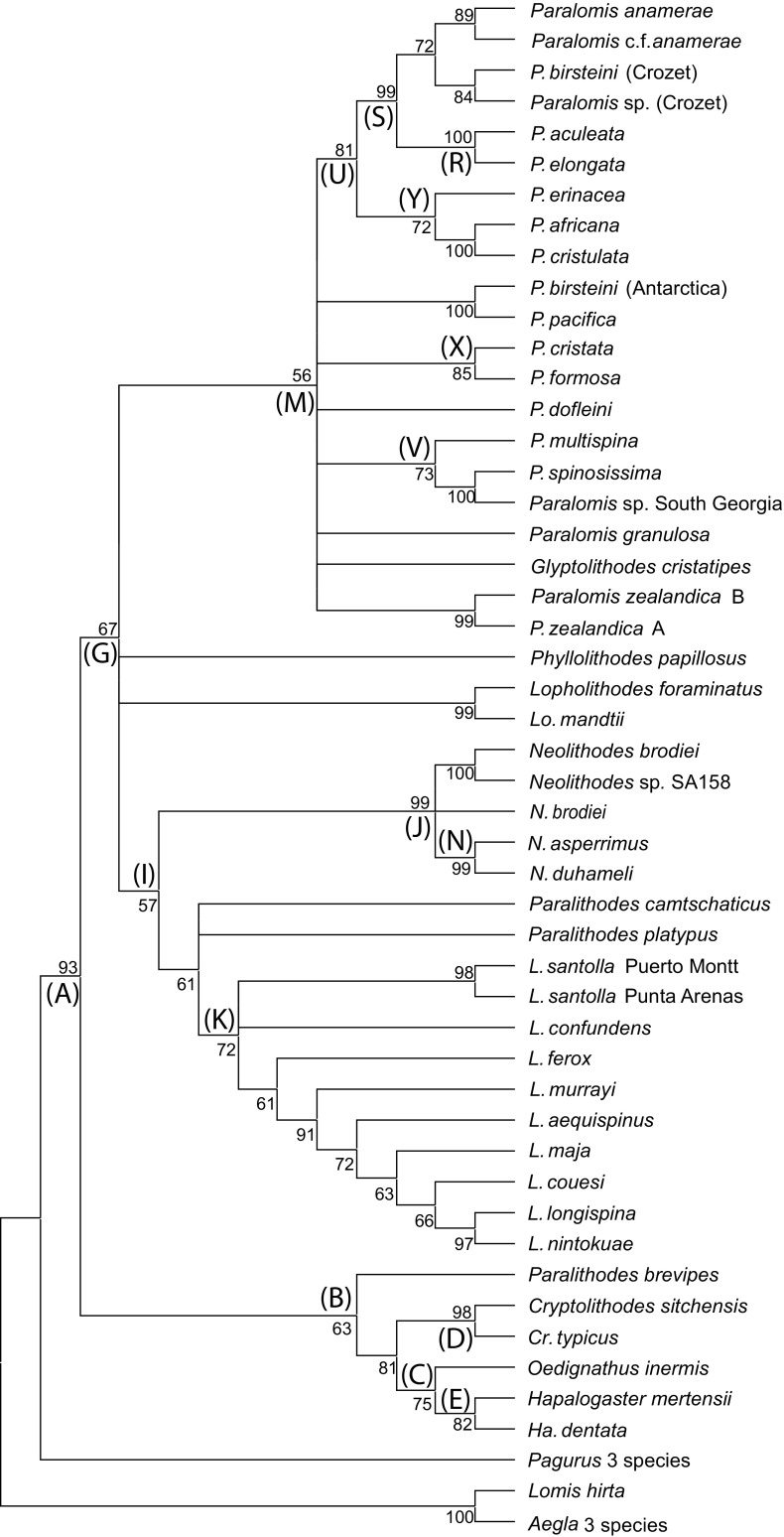
Trees produced from a total evidence alignment (TE_B_) of genes ITS1, 16S, B COI and 28SB. Terminal taxa are consensus sequences produced from multiple specimens using IUPAC ambiguity codes. Where sequences within species differ consistently at multiple loci species are split into multiple labels. Letters at nodes refer to clades discussed in the text. Minimum evolution tree produced in MEGA 3.1. Mid-point rooted. Numbers at nodes are bootstrap values (1000 replicates). Abbreviations: N = *Neolithodes*, L = *Lithodes*, P = *Paralomis*, Pa = *Paralithodes*, Lo = *Lopholithodes*, Cr = *Cryptolithodes*, Ha = *Hapalogaster*

**Fig. 4:**
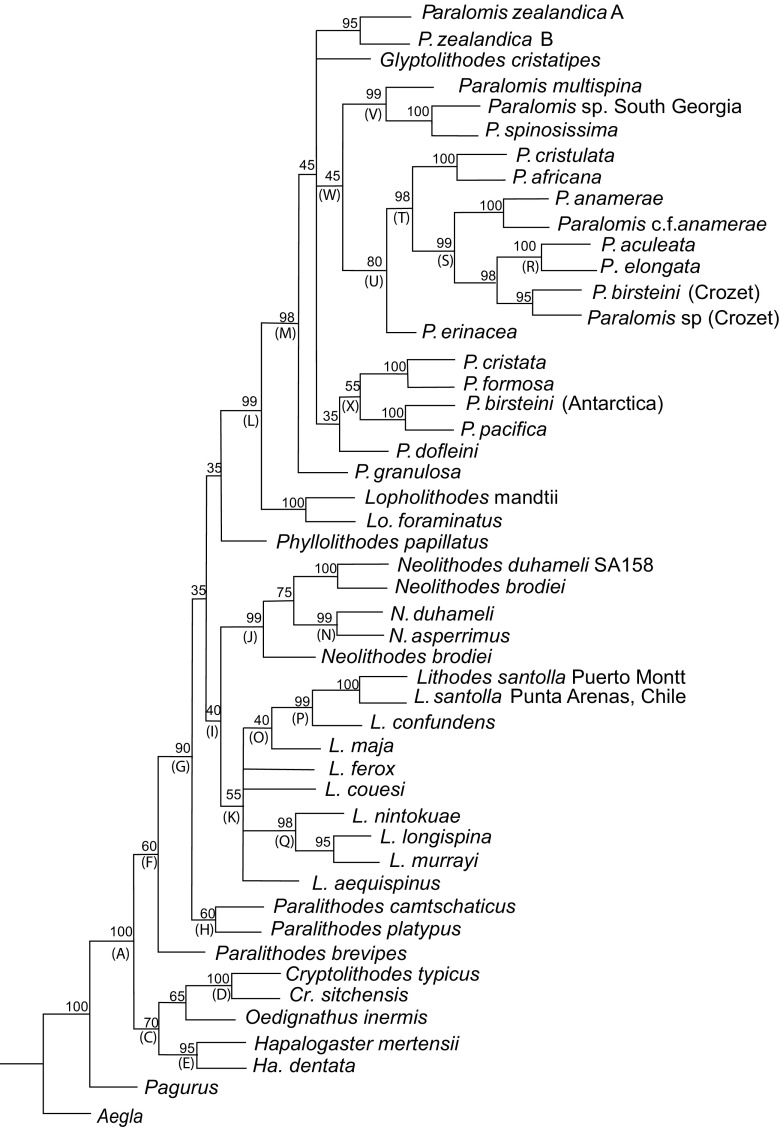
Trees produced from a total evidence alignment (TE_B_) of genes ITS1, 16S, B COI and 28SB. Terminal taxa are consensus sequences produced from multiple specimens using IUPAC ambiguity codes. Where sequences within species differ consistently at multiple loci species are split into multiple labels. Letters at nodes refer to clades discussed in the text. Maximum likelihood tree produced in PHYLIPDNAml. Rooted using a consensus sequence of three *Aegla* species. Numbers at nodes are bootstrap values (1000 replicates). Abbreviations: N = *Neolithodes*, L = *Lithodes*, P = *Paralomis*, Pa = *Paralithodes*, Lo = *Lopholithodes*, Cr = *Cryptolithodes*, Ha = *Hapalogaster*

**Fig. 5:**
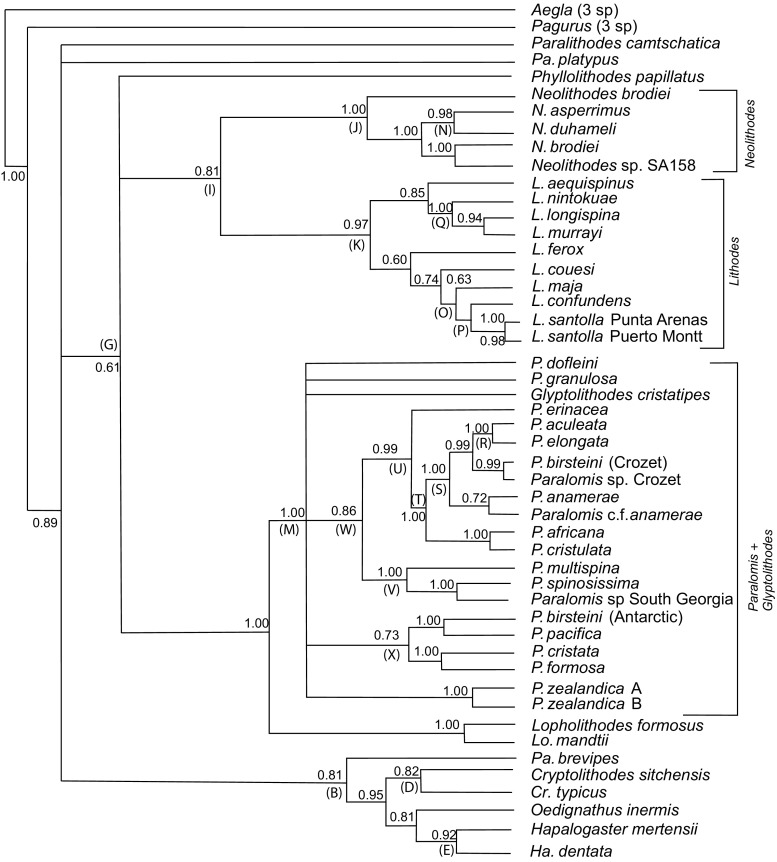
Trees produced from a total evidence alignment (TE_B_) of genes ITS1, 16S, B COI and 28SB. Terminal taxa are consensus sequences produced from multiple specimens using IUPAC ambiguity codes. Where sequences within species differ consistently at multiple loci species are split into multiple labels. Letters at nodes refer to clades discussed in the text. Tree produced by Bayesian analysis in MrBayes 3.1. Rooted using a consensus sequence of three *Aegla* species. Numbers at nodes are posterior probabilities. Abbreviations: N = *Neolithodes*, L = *Lithodes*, P = *Paralomis*, Pa = *Paralithodes*, Lo = *Lopholithodes*, Cr = *Cryptolithodes*, Ha = *Hapalogaster*

Genera *Cryptolithodes* [D] (98[100{0.82}]), *Hapalogaster* [E] (82[95{0.92}], *Oedignathus inermis*, and *Paralithodes brevipes* are excluded from a clade [G] (67[90{0.61}]) containing the remaining Lithodidae (Figs. 3, 4, 5). Relationships between these basal groups are ambiguous, because the ML, Bayesian and ME topologies do not agree whether the subfamily Hapalogastrinae [C] includes the (lithodine) genus *Cryptolithodes*. *Oedignathus* and *Hapalogaster* are sister taxa within clade [C] in the ME and Bayesian (51{0.81}) but not the ML topology, in which *Oedignathus* and *Cryptolithodes* are sister taxa [65]. *P. brevipes*, whilst being placed consistently outside a clade [G] uniting other lithodine genera, is placed alternately with the Hapalogastrinae [B] (63{0.81}) or at the base of the Lithodinae [F] [60] by different methods of analysis; this may suggest that *P. brevipes* belongs to a different genus. Monophyly of *Lithodes* [K] (55[72{0.97}]), *Neolithodes* [J] (99[99{1.0}]) and *Paralomis* (plus Glyptolithodes) [M] (98[56{1.0}]) are supported under all analytical methods. *Glyptolithodes* may indeed be synonymous with *Paralomis*. Support for a grouping of *Lithodes* with *Neolithodes* [I] (which is sometimes inclusive of *P. camtschatica* and *P. platypus*) is weak but present in all analyses (57[40{0.81}]). *Lopholithodes* is closely allied with *Paralomis* + *Glyptolithodes* [L] in the ML and Bayesian analyses [99{1.00}], and is never included within the monophyletic *Paralomis* (+*Glyptolithodes*) taxon [M]. The genus *Paralithodes* is represented by three species: *P. camtschatica*, *P. platypus* and *P. brevipes*. This genus is not supported as a monophyletic clade in any of the selected topologies. *P. platypus* and *P. camtschatica* are resolved as sister taxa in clade [H] under ML analysis [60]. *Neolithodes asperrimus* and *N. duhameli* are paired within clade [N] (99[99{0.98}]) to the exclusion of *N. brodiei*, and another Southern Pacific *Neolithodes* (species indeterminate) sample.

### *Lithodes*

Maximum likelihood analysis resolves two groups within *Lithodes*; separating *L. maja*, *L. santolla* and *L. confundens* [O] [40{0.63}] from *L. murrayi*, *L. longispina* and *L. nintokuae* [Q] (97[98{1.00}]). Within clade [O], South American species *L. santolla* and *L. confundens* are paired, forming clade [P] [99{1.00}] (Figs. 3, 4, 5). *L. ferox*, *L. couesi*, *L. maja*, and *L. aequispinus* can not be resolved with confidence on the basis of these results, instead appearing in a polytomy at the base of the *Lithodes* clade [K] in both the ME and ML analyses. In the Bayesian analysis, *L. aequispinus* is allied with *L. nintokuae*, *L. longispina* and *L. murrayi* outside clade [Q] {0.85}; and *L. ferox* and *L. couesi* outside the clade of Atlantic *Lithodes* [O].

### *Paralomis*

*Paralomis elongata* (paratype) is the sister group of *P. aculeata*, as indicated by clade [R] (100[100{1.00}]) (Figs. 3, 4, 5). There are no polymorphic sites in a comparison of 2110 bp of *P. elongata* and *P. aculeata* DNA. Clade [R] is nested within a larger clade of sub-Antarctic specimens [S] (99[99{1.00}]), including *P. anamerae* (caught by long-line fisheries in South Georgia); sample SA06 (morphological I.D. close to *P. anamerae*) in South Georgia; *P. birsteini* (sample SA147: Crozet); and *Paralomis* unidentified tissue sample (2 specimens: Crozet). An ‘African clade’ of *Paralomis* (*P. elongata*, *P. africana*, *P. cristulata*) is supported by ME analysis [Y] (72), but not by other optimisation criteria. ML analysis and Bayesian inference, produce topologies in which *P. africana* (+*P. cristulata*) are the sister taxa of clade [S] within a larger clade [T] [98{1.00}]. Clade [U] (Figs. 4, 5) includes *P. erinacea* as sister taxon to clade [T] with a high degree of support in the ML and Bayesian analyses [80{0.99}].

*Paralomis spinosissima* is the sister taxon of *Paralomis multispina* [V] (73[99{1.00}]), although the position of this clade is ambiguous within *Paralomis* as a whole. There is some indication that these species might be associated with clade [U], to form clade [W] [45{0.86}]. A derived relationship is indicated between *P. formosa* and *P. cristata* in clade [X] (85[100{1.00}]), as well as a similarity between an Antarctic specimen of *P. birsteini* and *P. pacifica* sequences from GenBank. Several other species, *Glyptolithodes cristatipes*, *P. dofleini*, *P. zealandica* and *P. granulosa*, are always included within *Paralomis* [M]; however, these species are never grouped with sufficient confidence alongside any of the other species included in the analysis. 64/1523 bp (4.2% of bases) are polymorphic when *P. birsteini* from the Crozet islands in the southern Indian Ocean, is compared with *P. birsteini* from the Ross Sea and Bellingshausen Sea (SA101 + SA85). These two strains of the *P. birsteini* morphotype do not form a monophyletic clade based on the genes sampled. In fact, fewer loci are polymorphic (1.57% of bases) when *P. birsteini* from Crozet is compared with *P. aculeata* (also from Crozet).

### Overview of phylogenetic signal from individual genes

The phylogenetic signal of the TE_B_ alignment (Fig. 3) appears to be derived predominantly from the COI gene, which has the highest level of sequence divergence within the group. Other genes support some of the very strong divisions (between genera or pairs of terminal taxa), but conflict exists at some of the intermediate nodes. Although there are gaps in species sampling and gene sampling, the topology of the tree should not change when more species are added, especially in well-supported groupings.

## Discussion

### Mutation rates within the Lithodidae

The number of polymorphic loci in the 5′ half of the 28S gene and the V4 region of the 18S gene was so low that the study of these genes was discontinued to economise on resources. A low level of sequence diversity within the Lithodidae is indicated; however, there is only incidental evidence to suggest that the expected levels of variation should be higher (Nelles et al. 1984; Crease and Taylor 1998; Held 2000). A like-with-like comparison of mutation rates between different lineages is almost impossible without geological calibration for the age of the taxon (which we do not have for the Lithodidae). For a homologous alignment of the 16S gene, a conservative estimate of expected amounts of variation was taken from monophyletic groups within the genera *Pagurus*, *Aegla* and *Emerita* (we emphasise the comparison of genus-level anomuran taxa with the whole subfamily Lithodinae). For 402 (in-group) bp of the 16S gene, the variation within a monophyletic clade of South American *Pagurus* was equal to that found within the whole lithodine subfamily, and 10 times the variation found within genus *Neolithodes*. The same degree of disparity was found within three species of *Emerita*. However, three species of *Aegla* had the same number of variable sites as the 15 tested species of the genus *Paralomis*. In the 18S gene, there were three variable positions between three Lithodine genera, but 11 within *Emerita* and 6 within *Aegla*. Disparity was less marked in the COI gene and comparisons were not made for 28S, COII or ITS. These results are not conclusive, but there is an indication that the Lithodinae have an atypically low genetic variation for such a large (and diverse) anomuran taxon of global distribution (Table 1). This could be evidence of a number of scenarios: a low rate of molecular evolution as a product of temperature-related change decreased enzymatic activity, decreased or less efficient DNA replication rates, and increased generation times experienced at low temperatures (Bargelloni et al. 1994; Martin 1999); alternatively, it could reflect a lineage-specific mutation rate that is not linked to low temperature physiology in ectotherms (Held 2001); a relatively recent radiation and/or a residual level of gene flow tending to increase the homogeneity of related species.

### Relationships between lithodid genera

**Fig. 6:**
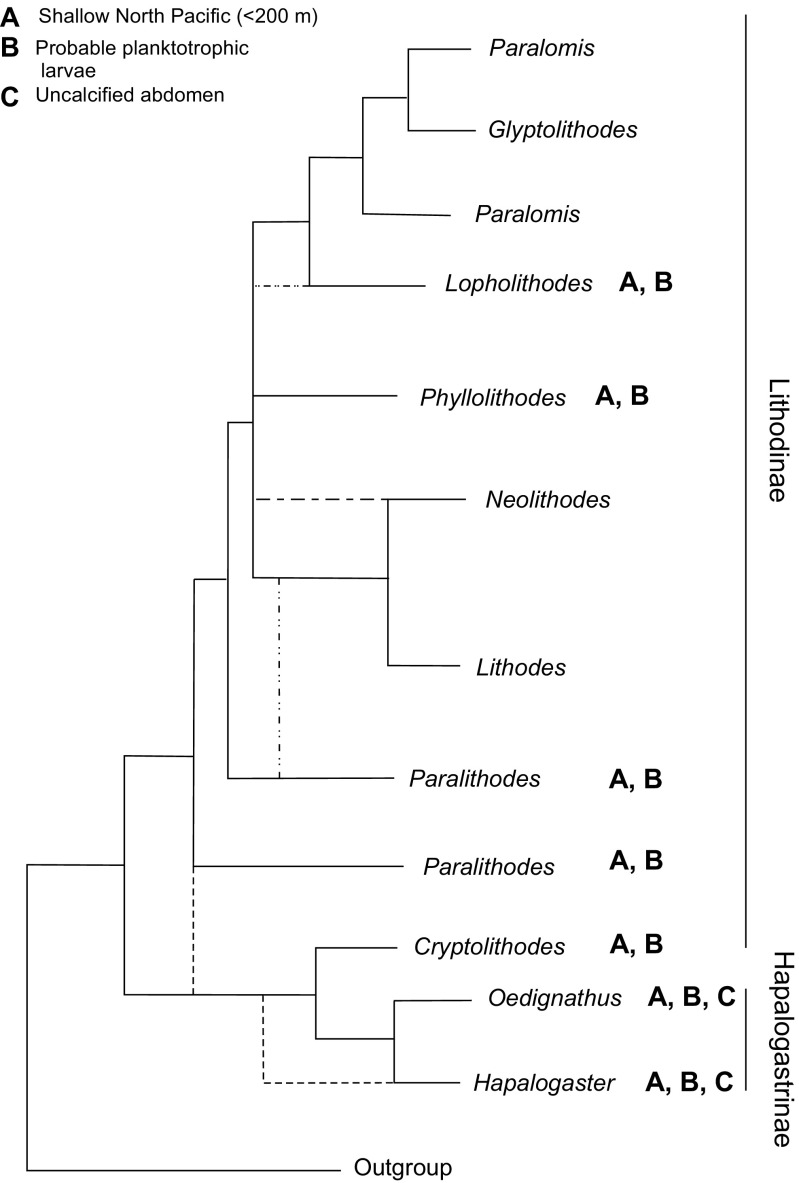
Schematic of the results of Minimum Likelihood (ML), Minimum Evolution (ME) and Bayesian (BAY) probability analyses for total evidence alignment TE_B_. In this tree, TE_C_, species within monophyletic genera are condensed to a single taxon label, and polyphyletic or paraphyletic genera are indicated as such by multiple taxon labels. Less frequent alternative topologies are indicated by dotted lines on the same tree

Of those studied, all lithodid genera are monophyletic as currently defined (McLaughlin et al. 2007, Bracken-Grissom et al. 2013, Noever and Glenner 2017), with the exception of *Paralithodes* (see below) and *Paralomis*, which include the single species of genus *Glyptolithodes*. *Paralomis*, *Glyptolithodes* and *Lopholithodes* are all lithodids with compact, well-calcified carapaces, and calcified plates on their abdominal segments (as opposed to nodules of calcification like in *Lithodes*, *Paralithodes* and *Neolithodes*; or uncalcified plates like those present in the Hapalogastrinae). *Lopholithodes* species *L. mandtii* and *L. foraminatus* diverge outside, but close to the base of the clade containing all sampled *Paralomis* and *Glyptolithodes* species. The arrangement of the genera does not provide conclusive evidence for or against the ‘hermit to king’ (Cunningham et al. 1992; Zaklan 2002a) or ‘king to hermit’ (McLaughlin and Lemaitre 1997) theories, nor was that the aim of the study. The data indicate that under the strict definition of the Lithodinae (including genus *Cryptolithodes*), the subfamily is probably paraphyletic (Figs. 3, 4, 5; see also Noever and Glenner 2017). This is a tentative confirmation of the evidence given by McLaughlin et al. (2004, 2007) that the soft abdomen of the Hapalogastrinae is not an ancestral feature (*Cryptolithodes* has a fully calcified abdomen with the fewest tergal plates of all Lithodidae); alternatively, *Cryptolithodes* could be secondarily calcified. Nevertheless, *Cryptolithodes* and the Hapalogastrinae belong to a lineage that diverged from the other Lithodinae at the base of the lithodid stem (see also Noever and Glenner 2017); these data indicate that a shallow water, North Pacific habitat and planktotrophic larval feeding mode are plesiomorphic features of the Lithodidae (Fig. 6; for discussion, see also Thatje and Hall 2016).

### The genus *Paralithodes*

There are six species included in the genus *Paralithodes*, three of which are sampled here: *P. brevipes*, *P. camtschatica*, *P. platypus*. The group is unified morphologically by having five plates rather than three on the second segment of the abdomen, and has no reduction of the antennal acicles. Small eggs (indicative of planktotrophic larval development) are shared between *P. brevipes*, *P. camtschatica*, *P. platypus* and several other North Pacific genera and are probably a plesiomorphic feature. Any derived morphological similarities between *P. camtschatica*, *P. brevipes* and *P. platypus* are contradicted by the evidence from their genetics that they are paraphyletic (see also Noever and Glenner 2017). Data intriguingly corroborate pre-cladistic theories developed by Bouvier (1895) and Makarov (1938) that predict the paraphyletic status of the genus *Paralithodes*, and specifically the closer relationship of *P. camtschatica* to *Lithodes* than to *P. brevipes*. The results of this analysis show that *P. camtschatica* and *P. platypus* are weakly allied with the *Lithodes* and *Neolithodes* genera.

### The genus *Neolithodes*

A markedly low within-genus mutation rate was observed for all genes sampled from specimens of *Neolithodes* (Online Supplementary Table 1). This suggests that either the three species sampled in this study are, by chance, particularly closely related within *Neolithodes*; or that these genes evolve more slowly in *Neolithodes* than they do in other genera. *N. asperrimus* occurs around the cape of Africa (from northern Namibia to Madagascar) and the Ivory coast (Macpherson 1988), at depths of 518–1050 m. Around 2000 km south of the cape, *N. duhameli* is known from the Crozet islands 620–1500 m. All three of these species are characterised within *Neolithodes* by having a large number of spinules on the carapace and all appendages. It is notable that of these three species, the two that are geographically closest are the most closely related.

### The genus *Lithodes*

The structure of the *Lithodes* clade is not well resolved. Analysis of the same dataset produces a number of contradicting topologies. *L. murrayi* and Pacific species *L. nintokuae* and *L. longispina* are typically separated from the Atlantic species *L. santolla*, *L. confundens*, *L. ferox* and *L. maja*. *L. murrayi* shares a morphological affinity with *Lithodes* species from the North-west Pacific and Indian Oceans (*L. longispina*, Macpherson and Chan 2008, *L. richeri*), rather than with those from the North East Pacific or Atlantic (*L. aequispinus*, *L. santolla*, Macpherson 1988). *L. murrayi* is, however, currently considered to have a wide distribution around the Southern Ocean: reported from Crozet (as sampled here), as well as the Bellingshausen Sea; however, it is not known from New Zealand and Australia (see Garcia-Raso et al. 2005; Ahyong 2010; Anosov et al. 2015), and morphological similarities are strong with *L. turkayi* from the Scotia Sea. *L. ferox* is found along the western coast off Africa, and shares a number of morphological features with *L. murrayi*. The separation of *L. murrayi* and *L. ferox* suggests that the common ancestor of these two groups possessed morphological characteristics that these two now share. It is noteworthy that *L. maja* is placed close to *L. santolla* and *L. confundens* in the TEB ML and Bayesian trees, since these are all Atlantic species from northern and southern high latitudes, respectively.

*Lithodes santolla* is morphologically similar to *L. confundens*, primarily based on the absence of a prominent mid-rostral spine and this feature distinguishes them from many in the genus (Macpherson 1988). The distinction between the species is based on the number and size of spines on the dorsal carapace and articles of the walking legs (which are larger and fewer in *L. santolla*), and these two species have an overlapping distribution in and around Patagonia (South America) (Lovrich et al. 2002; Perez-Barros et al. 2015). Information obtained in this study shows a consistent genetic difference between individuals belonging to the two species. There also appears to be two ‘haplotypes’ of *L. santolla*: one from individuals sampled around Puerto Montt (west coast Chile, 40° S), and one from individuals sampled at Punta Arenas (Straits of Magellan). The two strains of *L. santolla* are more closely related to one another than either is to *L. confundens* (for discussion, also see Perez-Barros et al. 2015).

The Straits of Magellan consists of several basins with hydrological and geological boundaries between them (Brambati et al. 1991). In several places, shallow sills constrain water exchange to the upper 40 m; it is possible that there is some restriction in the level of gene flow between the shallow water (10–212 m) populations of *L. santolla* from the West Coast and those in the central parts of the Strait of Magellan (Antezana 1999). Samples of *L. confundens* were obtained from two locations (unfortunately neither of them the same as *L. santolla* sample sites): Cabo San Sebastian (0–10 m), on the Eastern coast of Argentina, and further (and deeper: 162 m) off shore on the Argentinean Plateau.

### The genus *Paralomis*

At least 61 species (including *G. cristatipes*) are currently recognised from within the genus *Paralomis*. Molecular data have been obtained for 16 of these species, broadly representing the total distribution of the genus throughout most of the world’s oceans. The phylogenetic positioning of *Glyptolithodes* may indeed suggest that *Glyptolithodes* is synonymous with *Paralomis.* Ancestral relationships at the base of the *Paralomis* clade have proved difficult to reconstruct based on these data. Pacific species *P. zealandica*, *G. cristatipes*, *P. dofleini*, and South Atlantic species *P. granulosa* in particular are not close enough to any other sampled species for ancestral similarities to be recognised. This perhaps reflects gaps in sampling in the Pacific region, which is known to have a high morphological diversity of Lithodidae. From this basal polytomy, two or three larger clades are resolved [V, U, X] or [W, X] (Figs. 3, 4, 5). Clade [X] contains an apparent assortment of North Pacific and South Atlantic *Paralomis*, and strongly implies a relationship between *P. formosa* and *P. cristata* that does not seem to reflect a distinct morphological or distributional similarity.

West African species *P. erinacea*, *P. africana* and *P. cristulata* (Macpherson 1988) diverge at the base of a second clade, [U] (Figs. 3, 4, 5). The recognised ranges of *P. africana* and *P. cristulata* have been recently expanded by explorations off the coast of Mauritania (courtesy of Ana Ramos, Vigo) and videos from the Serpent projects off Nigeria (courtesy of Dan Jones, NOCS). Crabs with morphologies similar to *P. africana* and *P. cristulata* are now known from locations all along the coast around equatorial western Africa, around 1366 m deep off Nigeria, and 1500 m off Mauritania (Macpherson 1988). This extends the previously recorded southern distribution of *P. africana* substantially. It also means that the two groups have an adjacent, if not overlapping, distribution in this area. Molecular evidence supports a very close genetic relationship between these species, which can be distinguished by the form of lateral spines on the legs and carapace. These appear as strong crests in *P. cristulata* but several spines in *P. africana* (Macpherson 1988a). *P. erinacea* has a broadly overlapping range with *P. cristulata*, and was found alongside this species in Mauritania. It is morphologically quite different to the two other African species: most notably in the presence of spines uniformly covering the carapace, each with many long setae around the mid portion of the spines (Hall and Thatje 2010). The original description (Macpherson 1988) states that *P. erinacea* is close to *P. spinosissima* from the South Atlantic, with its carapace covered uniformly in large spines. *P. erinacea*, however, does not have an obviously enlarged spine in the mid part of its gastric region, which (among other features) distinguishes it from *P. multispina* and *P. spinosissima*. In addition, the setae on the spines of *P. erinacea* are of a substantially different form to those in the later species (Hall and Thatje 2010). A consistently well resolved part of the tree, within clade [U] (Figs. 3, 4, 5) contains specimens exclusively from the sub-Antarctic region of the Atlantic and Indian Oceans at latitudes above 45° S [S]. *P. anamerae*, *P. aculeata* and *P. elongata* have distributions around isolated islands and seamounts at the latitudes associated with the eastward flowing Antarctic circumpolar current. The close genetic relationship between these groups suggests that they are not as isolated as their patchy distribution would suggest. No monophyletic group exists containing all southern high latitude *Paralomis* species; *P. formosa*, *P. granulosa* and *P. spinosissima* from sub-Antarctic waters, which all resolved elsewhere on the tree.

Known from opposite ends of the globe, *P. spinosissima* and *P. multispina* (clade [V]) certainly do not have an adjacent distribution. The tissue sample of *P. multispina* used in this study was taken from a preserved specimen found in waters off Japan (and also sequences from GenBank, Online Supplementary Table 1), although the species is known throughout the Bering Sea, and from the coast of North America between 600 and 1500 m. *P. spinosissima* is known from the Scotia Sea and the South Atlantic, as well as from waters south off Cape Horn (162–1200 m), with distribution skewed towards the shallower end of this range (Purves et al. 2003). The evidence presented here suggests a close ancestral relationship between the two species.

*Paralomis multispina* and *P. spinosissima* have a uniform coverage of spines across the carapace and legs, which in adults have an oblique face at the apex, with a ring of setae around the tip (Hall and Thatje 2010). They both have 3–5 large, pointed spines without setae emanating from the gastric and branchial regions, and also from the lateral margins and legs. Tissue samples from preserved specimens of *P. phrixa* from the western coast of Peru (815–860 m) did not yield any good quality DNA, and no genetic data were obtained for this species. *P. phrixa* does, however, have spines and aspects of morphology of a similar form to *P. spinosissima* and *P. multispina*. Its intermediate distribution bridges the geographic gap between the North Pacific and the South Atlantic and implies a radiation of this group along the western coast of the American continent.

### Spines: Morphological classifications

Presence or absence of a continuous coverage of spines is an obvious morphological trait by which *Paralomis* can be classified (Hall and Thatje 2010). There are several heavily spined *Paralomis* species in the global oceans, only three of which have been sampled for the genetic study, although most have been examined for morphological traits. *P. bouvieri*, *P. hystrix*, and *P. aspera* (and similar species *P. makarovi*—described Hall and Thatje 2009a) are all distinguished based on many aspects of morphology, including spine form on a microscopic level (Hall and Thatje 2010). If *P. erinacea* does have a monophyletic relationship with *P. anamerae* to the exclusion of *P. spinosissima* [U], then either the common ancestor of this lineage [W] was covered in spines, or the condition has arisen separately in at least two lineages (Figs. 3, 4, 5).

#### *Paralomis granulosa*

On the strength of evidence from many genes, *P. granulosa* is distantly related to all of the other 15 *Paralomis* species examined in the genetic study. The Magellanic fauna is thought to be relatively young, as until recently (approx. 10,000 years) the area was glaciated and had no marine influence (McCulloch et al. 2000; Hulton et al. 2002). *P. granulosa* is also known from the Falkland islands and from deeper waters (100–150 m) between the Falkland islands and the Straits of Magellan; however, it shares neither strong morphological nor genetic links, with any of the currently known Scotia Sea *Paralomis*. No west coast *Paralomis* species were sampled for this study, but it might be hypothesised that the ancestral links of *P. granulosa* are with deeper (> 400 m) *Paralomis* on the continental slopes of central Chile, where a wide variety of species are known.

#### *Paralomis birsteini*

Specimens of *P. birsteini* have been sampled from populations in the Ross Sea, Bellingshausen Sea, and southern Indian Ocean (Crozet islands). These specimens conform to the original species description (Macpherson 1988a; Ahyong and Dawson 2006), based on personal examination of 10 specimens covering these three locations. Analysis of the COI, 16S, 28S and ITS genes in the a Bellingshausen Sea specimen of *P. birsteini* (SA101) (Thatje et al. 2008) and a Ross Sea specimen (SA85), in comparison with several Crozet specimens suggest that gene flow within this morphotype is limited or absent between the Southern Ocean and Crozet populations. Cryptic speciation has been previously discovered in other Antarctic taxa with limited dispersal potential (Held and Wägele 2005; Raupach and Wägele 2006). However, the level of variation between these two populations of *P. birsteini* seems to surpass a cryptic speciation event, and within the context of the global diversity of genus *Paralomis*, it seems that these populations are not closely related. The morphological similarities between Indian Ocean (Crozet) and Southern Ocean (Bellingshausen Sea, Ross Sea: similar to one another) examples of *Paralomis* are not reflected in genetic markers. It is possible that these populations have been mistakenly unified as a species, and are in fact similar by convergence. Otherwise, a study including greater sample numbers from each of these populations might be able to identify multiple genotypes at each location (for biogeography, see also Aronson et al. 2015).

## Future work

There are indications of lower than expected levels of mutation within the Lithodidae and a thorough investigation of this phenomenon will be proposed for further work. This could indicate a recent common ancestor to the extant group or a slow rate of molecular evolution in the Lithodidae. The Lithodinae as defined to include North Pacific genus *Cryptolithodes* may be paraphyletic, with the Hapalogastrinae and *Cryptolithodes* as sister taxa. This implies that the soft-bodied abdomen of the Hapalogastrinae might not be plesiomorphic for the Lithodidae (see also Noever and Glenner 2017); however, this aspect will require further investigation. Several species of *Paralomis* do not resolve consistently with any other groups sampled, implying a complex and possibly rapid global evolution early in the history of the genus; the genus may require extensive revision using more taxa.

## Electronic supplementary material

ESM 1 (DOCX 69 kb).
