# Supplementary material for: Evolution through cold and deep waters: the molecular phylogeny of the Lithodidae (Crustacea: Decapoda)
Source: Naturwissenschaften. 2018 Feb 27;105(3):19. doi: 10.1007/s00114-018-1544-2 (PMC5829116; doi:10.1007/s00114-018-1544-2)
Supplement: Supplementary file 1 — (DOCX 69 kb). [file 114_2018_1544_MOESM1_ESM.docx]

**Online Supplementary Material**

**Evolution through cold and deep waters: the molecular phylogeny of the Lithodidae (Crustacea: Decapoda)**

Sally Hall^1^, Sven Thatje^1^ *

^1^Ocean and Earth Science, University of Southampton, National Oceanography Centre, Southampton, European Way, Southampton, SO14 3ZH, United Kingdom

*Corresponding author

*E-mail address: svth@noc.soton.ac.uk

**Online Supplementary Table 1.** Taxa included in this study with list of GenBank and BOLD accession numbers of the molecular markers used. N/S = non-submitted to GenBank because of an exact duplicate of other (above) specimen. Museum collection number assigned where available; otherwise, samples are from research cruises and in private collection (S. Hall). ICMD= Muséum national d’Histoire naturelle, Paris; SKG= Senckenbergmuseum Frankfurt; GBK= from GenBank (GenBank 2017).

| **Taxon** | Sample location  (collection) | **COI** | **16S** | **ITS1** | **28SB** |
| --- | --- | --- | --- | --- | --- |
| **Family Lithodidae** |  |  |  |  |  |
| *Neolithodes asperrimus* | Mauritania | HM020891 | HM020938 | HM021016 | - |
| *Neolithodes asperrimus* | Mauritania | HM020890 | HM020937 | - | - |
| *Neolithodes asperrimus* | Mauritania | - | N/S | HM021019 | - |
| *Neolithode asperrimus* | Mauritania | - | N/S | HM021018 | HM020848 |
| *Neolithode asperrimus* | Mauritania | - | - | HM021020 | - |
| *Neolithode asperrimus* | Mauritania | - | N/S | HM021017 | HM020847 |
| *Neolithodes sp* | Crozet Island | HM020895 | HM020948 | - | - |
| *Neolithodes brodiei* | New Zealand | HM020893 | HM020944 | - | HM020851 |
| *Neolithodes brodiei* | New Zealand | HM020894 | FJ462644 | - | HM020852 |
| *Neolithodes brodiei* | New Zealand | EU493263 | HM020942 | - | - |
| *Neolithodes brodiei* | New Zealand | N/S | HM020943 | - | HM020853 |
| *Neolithodes duhameli* | Crozet Island | N/S | HM020945 | HM021021 | HM020849 |
| *Neolithodes duhameli* | Crozet Island | HM020896 | HM020946 | HM021022 | HM020850 |
| *Paralithodes brevipes* | Japan  (GBK) | AB211297.1  AB211298.1  AB211299.1  AB211300.1 | AF425337.1 | - | AB211308.1 |
| *Paralithodes platypus* | Japan  (GBK) | AB211448.1  AB211301.1  AB211302.1  AB211447.1  AB211444.1 | - | - | AB193822.1  AB193821.1 |
| *Paralithodes camtschaticus* | Japan  (GBK) | AF425376 | AF425338.1 | - | AB193824  AB193823 |
| *Lithodes longispina* | Japan  (GBK) | AB476813  AB476814  AB476815 | - | - | - |
| *Lithodes aequispinus* | (GBK)  Japan | AF425308 | - | - | - |
| *Lithodes couesi* | Canada: British Columbia (GBK) | DQ882086  DQ882085 | - | - | - |
| *Lithodes maja* | Canada: Gulf of St. Lawrence (GBK) | FJ581742  FJ581745 | AF425330.1 | - | - |
| *Lithodes santolla* | Chile, Puerto Montt | HM020897 | HM020955 | HM021015 | HM020861 |
| *Lithodes santolla* | Chile, Punta Arenas | - | AY595927.1 | - | AY596100 |
| *Lithodes santolla* | Chile, Punta Arenas | - | AY595927.1 | - | AY596100 |
| *Lithodes santolla* | Chile, Punta Arenas | HM020898 | - | - | HM020859 |
| *Lithodes santolla* | Argentina: Beagle Channel (GBK) | - | AF425331.1 | - | - |
| *Lithodes nintokuae* | Central north Pacific  (GBK) | AB375135  AB375146  AB375137 | - | - | - |
| *Lithodes murrayi* | Crozet Island | - | - | HM021014 | - |
| *Lithodes murrayi* | Crozet Island | HM020899 | HM020953 | HM021012 | HM020857 |
| *Lithodes murrayi* | Crozet Island | HM020899 | HM020953 | HM021013 | HM020858 |
| *Lithodes confundens* | Argentina, southwest Atlantic | EU493257 | EU493273 | - | - |
| *Lithodes confundens* | Argentina, southwest Atlantic | HM020901 | HM020949 | HM021008 | - |
| *Lithodes confundens* | Chile | HM020900 | FJ464648 | - | HM020855 |
| *Lithodes ferox* | Namibia  (ICMD 331/2000) | HM020903 | HM020952 | HM021009 | HM020856 |
| *Lithodes ferox* | Namibia  (ICMD 112/1991) | N/S | HM020950 | N/S | - |
| *Lithodes ferox* | Mauritania | N/S | HM021011 | N/S | - |
| *Lithodes ferox* | Mauritania | N/S | HM020951 | HM021010 | - |
| *Paralomis aculeata* | Crozet Island | HM020904 | HM020957 | HM020984 | HM020862 |
| *Paralomis aculeata* | Crozet Island | HM020904 | HM020958 | HM020985 | HM020862 |
| *Paralomis aculeata* | Crozet Island | HM020904 | - | HM020983 | N/S |
| *Paralomis anamerae* | South Georgia | HM020906 | HM020960 | HM020987 | HM020865 |
| *Paralomis anamerae* | South Georgia | HM020905 | HM020959 | - | N/S |
| *Paralomis anamerae* | South Georgia | HM020905 | HM020959 | - | HM020866 |
| *Paralomis anamerae* | South Georgia | HM020906 | - | - | - |
| *Paralomis africana* | Namibia  (ICMD 302/2000) | HM020907 | - | - | HM020864 |
| *Paralomis africana* | Namibia  (ICMD 81/1991) | - | EU493275 | - | - |
| *Paralomis cristulata* | Guinea  (ICMD 130/1991) | HM020908 | - | - | HM020870 |
| *Paralomis birsteini* | Western Antarctic Peninsula | EU493260 | HM020988 | HM020988 | HM020867 |
| *Paralomis birsteini* | Scott Island, Ross Sea | HM020909 | N/S | - | - |
| *Paralomis birsteini* | Kerguelen Island | HM020910 | - | - | HM020868 |
| *Paralomis indet* | Crozet Island | N/S | HM020961 | - | - |
| *Paralomis indet* | Crozet Island | N/S | N/S | - | HM020869 |
| *Paralomis cristata* | Japan | HM020911 | EU493267 | - |  |
| *Paralomis dofleini* | Japan  (SKG 31816) | HM020912 | - | - | HM020871 |
| *Paralomis dofleini* | Japan  (SKG 31816) | HM020913 | HM020962 | - | - |
| *Paralomis elongata* | Bouvet Island | HM020914 | HM020872 | HM020989 | HM020872 |
| *Paralomis erinacea* | Mauritania | HM020917 | HM020965 | HM020991 | HM020873 |
| *Paralomis erinacea* | Mauritania | - | HM020966 | HM020992 | HM020873 |
| *Paralomis erinacea* | Mauritania | - | HM020967 | HM020993 | HM020873 |
| *Paralomis erinacea* | Mauritania | HM020915 | N/S | HM020994 | HM020873 |
| *Paralomis erinacea* | Mauritania | - | HM020963 | HM020995 | HM020873 |
| *Paralomis formosa* | South Georgia | HM020919 | HM020973 | HM020998 | HM020875 |
| *Paralomis formosa* | South Georgia | HM020918 | N/S | - | - |
| *Paralomis formosa* | South Georgia | HM020920 | HM020972 | HM020999 | HM020874 |
| *Paralomis formosa* | South Georgia | HM020921 | HM020974 | HM021000 | - |
| *Paralomis formosa* | South Georgia | HM020922 | HM020975 | - | - |
| *Paralomis formosa* | South Georgia | EU493262 | FJ462645 | - | HM020876 |
| *Paralomis formosa* | South Georgia | - | HM020970 | HM021001 | - |
| *Paralomis anamerae* | South Georgia | N/S | N/S | - | - |
| *Paralomis granulosa* | Chile, Punta Arenas | - | AF425339.1 | - | - |
| *Paralomis granulosa* | Chile, Punta Arenas | EU493264.1  HM020925 | HM020976 | HM021004 | - |
| *Paralomis granulosa* | Chile, Punta Arenas | - | - | HM021003 | HM020877 |
| *Paralomis granulosa* | Chile, Punta Arenas | EU493264  HM020926 | EU493274.1 | HM021002 | - |
| *Paralomis granulosa* | Chile, Punta Arenas | - | EU493278.1 | - | - |
| *Paralomis spinosissima* | South Georgia | EU493259 | HM020982 | - | - |
| *Paralomis spinosissima* | South Georgia | EU493258 | N/S | - | - |
| *Paralomis spinosissima* | South Georgia | HM020927 | - | - | HM020879 |
| *Paralomis spinosissima* | South Georgia | HM020928 | EU493259 | - | HM020880 |
| *Paralomis spinosissima* | South Georgia | HM020931 | N/S | HM021007 | - |
| *Paralomis spinosissima* | South Georgia | HM020932 | N/S | HM021005 | - |
| *Paralomis spinosissima* | South Georgia | HM020933 | N/S | HM021006 | - |
| *Paralomis pacifica* | Pacific Ocean  (GBK) | AB476747  AB476748  AB476749 | - | - | - |
| *Paralomis multispina* | Japan  (SKG 30854) | N/S | - | - | - |
| *Paralomis multispina* | Japan  (GBK) | AB375545  AB428440  AB428440  AB428437 | - | - | - |
| *Paralomis zealandica* | New Zealand | HM020935 | HM020981 | - | - |
| *Paralomis zealandica* | New Zealand | HM020936 | HM020980 |  |  |
| *Paralomis zealandica* | New Zealand | N/S | N/S | - | - |
| *Lopholithodes foraminatus* | Canada: British Columbia  (GBK) | DQ882089.1  DQ882087.1 | - | - | - |
| *Lopholithodes mandtii* | Canada: British Columbia  (GBK) | AF425372 | AF425333.1 | - | - |
| *Phyllolithodes papillosus* | Canada: British Columbia  (GBK) | AF425378 | AF425340.1 | - | - |
| *Cryptolithodes sitchensis* | Canada: British Columbia  (GBK) | AF425363 | AF425324.1 | - | - |
| *Cryptolithodes typicus* | Canada: British Columbia  (GBK) | AF425364 | AF425325.1 | - | - |
| *Glyptolithodes cristatipes* | California  (GBK) | AF425365 | AF425365 | - | - |
| *Hapalogaster mertensi* | Canada: British Columbia  (GBK) | AF425367 | AF425328.1 | - | - |
| *Hapalogaster dentata* | Japan  (GBK) | AF425366 | AF425366 | - | - |
| *Oedignathus inermis* | California  (GBK) | EU329164.1 | - | - | - |
| *Oedignathus inermis* | Canada: British Columbia  (GBK) | AF425373 | AF425334.1 | Z14062.1 | - |
| **Family Lomisidae** |  |  |  |  |  |
| *Lomis hirta* | (GBK) | AY595672.1  AF436035.1 | AY595928.1  AF436052.1 | - | AF435993.1 |
| **Family Aeglidae** |  |  |  |  |  |
| *Aegla intercalata* | (GBK) | AY595666.1  AY595665.1  AY595664.1 | AY595920.1  AY595919.1  AY595918.1 | - | AY596091.1  AY596090.1  AY596089.1 |
| *Aegla neuquensis* | (GBK) | AY595668.1 | AY595922.1  AY595921.1 | - | AY596093.1  AY596092.1 |
| *Aegla platensis* | (GBK) | AY595663.1  AY595662.1  AY595644.1  AY595643.1 | AY595917.1  AY595916.1  AY595898.1  AY595897.1 | - | AY596088.1  AY596087.1  AY596069.1  AY596068.1 |
| **Family Paguridae** |  |  |  |  |  |
| *Pagurus comptus* | (GBK) | - | FJ869144.1  FJ869142.1  FJ869145.1 | - | - |
| *Pagurus pollicaris* | (GBK) | AF483171.1  AF483170.1  AF483169.1 | FJ869152.1  U96089.1 | - | - |
| *Pagurus longicarpus* | (GBK) | FJ581826.1  FJ581825.1  FJ581824.1  FJ581823.1 | AF150756.1 | - | AY739185.1  NC003058.1 |
| *Pagurus bernhardus* | (GBK) | AF483157.1 |  |  |  |
| *Pagurus brevidactylus* | (GBK) | - | DQ369945 | - | - |
| *Pagurus leptonyx* | (GBK) | - | DQ369946.1 | - | - |
| **Family Hippidae** |  |  |  |  |  |
| *Emerita analoga* | (GBK) | L43101.1  L43099.1 | L43108.1  AF246154.1  AF246153.1  L43107.1 | - | - |
| *Emerita brasiliensis* | (GBK) | L43151.1 | DQ079712.1  L43110.1 | - | - |
| *Emerita talpoida* | (GBK) | L43106.1  L43105.1  L43104.1 | AF246152.1  AF246151.  AF246150.1 | - | - |

**Supplementary Reference**

GenBank (2017) accessed 19/08/17; <https://www.ncbi.nlm.nih.gov/genbank/>
